# Differential β-catenin expression levels are associated with morphological features and prognosis of colorectal cancer

**DOI:** 10.3892/ol.2014.2433

**Published:** 2014-08-11

**Authors:** ZHAO-HUA GAO, CHONG LU, MEI-XIAN WANG, YI HAN, LI-JUAN GUO

**Affiliations:** 1 Department of Surgical Oncology and General Surgery, First Hospital of China Medical University, Shenyang, Liaoning 110001, P.R. China; 2 Department of Surgery, Shenyang Red Cross Hospital, Shenyang, Liaoning 110013, P.R. China; 3 Department of Breast Surgery, Liaoning Province Cancer Hospital and Institute, Shenyang, Liaoning 110042, P.R. China; 4 Department of Tumor Pathology and Surgical Oncology, First Hospital of China Medical University, Shenyang, Liaoning 110001, P.R. China

**Keywords:** β-catenin, colorectal cancer, epithelial-mesenchymal transition, tumor budding, invasive front

## Abstract

β-catenin, an epithelial-mesenchymal transition (EMT)-associated marker, is key in the progression of colorectal cancer (CRC). However, the prognostic significance of β-catenin expression in patients with CRC remains controversial. In the present study, the expression of β-catenin at the tumor invasive front and the tumor center was investigated, and the correlations amongst β-catenin differential expression patterns and the clinicopathological characteristics and prognosis of CRC patients were determined. In total, 181 patients that were diagnosed with CRC (as determined by histopathological evaluation) and subjected to surgical resection at the First Hospital of China Medical University between 2000 and 2001 were examined, and CRC specimens were obtained. Immunohistochemical (IHC) staining of β-catenin was performed for each specimen. The nuclear β-catenin expression levels were identified to be significantly lower in the tumor center than at the tumor invasive front (immunoreactivity score, 0.05±0.303 versus 2.18±3.917; P<0.001). The presence of nuclear β-catenin overexpression at the tumor invasive front was found to be correlated with the tumor, node, metastasis stage (P=0.020), lymph node metastasis (P=0.016) and histological differentiation (P=0.006). Survival analysis revealed that reduced membranous expression levels and increased nuclear expression levels of β-catenin were statistically significantly associated with poor survival times. Furthermore, differential β-catenin expression levels were associated with aggressive morphological features, EMT and a poor prognosis in CRC. Therefore, IHC analysis of β-catenin is considered to be a useful marker to predict the prognosis in patients with CRC.

## Introduction

Colorectal cancer (CRC) is the second most common cause of cancer-associated mortality worldwide. Overall, ~50% of patients diagnosed with CRC succumb to the disease, due to complications associated with distant metastasis (1). The incidence of CRC in China has increased over recent years, particularly in the more developed areas (2). The tumor-host interaction at the invasive margin of CRC is a crucial interface where tumor progression and tumor cell dissemination ensue (3). Epithelial-mesenchymal transition (EMT), a dynamic process of colorectal carcinoma cell dedifferentiation, occurs at the invasive tumor front (4). The biological behavior of cancer is considered to be more accurately reflected by the histologic features present at the invasive front rather than those observed at the tumor center.

β-catenin is a component of the Wingless/Wnt signaling pathway. Dysfunction of the Wnt signaling pathway is important in colorectal carcinogenesis and results in the nuclear accumulation of β-catenin (5). Membranous beta-catenin forms a complex with E-cadherin, a critical mediator of cell-cell adhesion, and is responsible for the maintenance of cell polarity (6). The membranous expression of beta-catenin and E-cadherin characterizes the epithelial phenotype whereas the loss of this membranous expression is indicative of a switch toward a more mesenchymal phenotype. The nuclear translocation of β-catenin induces EMT and pro-invasive gene expression (7). Therefore, the differential intracellular distribution of β-catenin exerts a marked impact on the phenotype and behavior of tumor cells (8).

In the present study, the expression of the EMT-associated marker, β-catenin at the tumor invasive front and tumor center was investigated using immunohistochemical (IHC) analysis, and the correlations among β-catenin differential expression patterns, and clinicopathological characteristics and prognosis in CRC cases were determined.

## Materials and methods

### Patients and specimens

A total of 181 CRC tissue samples were obtained from patients diagnosed with CRC (as determined by histopathological evaluation) and subjected to surgical resection at the First Hospital of China Medical University (Shenyang, China) between 2000 and 2001. None of the patients had been treated with preoperative chemotherapy or radiation. Two senior pathologists reviewed the tissue sections from all of the cases. Tumor histological classification was assessed according to the World Health Organization criteria (9) and classified using the seventh edition of the tumor, node, metastasis (TNM) staging manual produced by the International Union Against Cancer/American Joint Committee on Cancer (2010) (10). All patients were followed up via telephone enquiry or questionnaire. The follow-up time ranged between 1.5 and 71 months (median, 51 months). The Ethics Committee of China Medical University approved the use of tissue samples in this study. Written informed consent was obtained from all of the patients.

### Antibodies and reagents

The primary antibodies used were monoclonal rabbit anti-human β-catenin (Abcam, Cambridge, UK).

### Immunohistochemistry

Formalin-fixed, paraffin-embedded sections (4-μm thick) were prepared from the CRC samples. The tissue sections were deparaffinized and rehydrated via sequential washing with xylene, graded ethanol and phosphate-buffered saline (PBS). Following deparaffinization and rehydration, the tissue sections were subjected to high temperature-induced epitope retrieval by briefly steaming in target retrieval solution (10 mM citrate buffer; pH 6.0; (Beijing Zhongshan Golden Bridge Biotechnology Co., Ltd., Beijing, China). Subsequently, the sections were treated with normal goat serum blocking solution (Beijing Zhongshan Golden Bridge Biotechnology Co., Ltd.) and then incubated with β-catenin primary antibody (dilution, 1:500) overnight at 4°C. Antibody binding was detected using an SP reagent kit (Zhongshan Chemical, Beijing, China) following the manufacturer’s instructions. PBS replaced the primary antibody in the negative control and samples that were known to express β-catenin served as the positive controls. All sections were counterstained with hematoxylin, dehydrated and mounted.

### Evaluation of staining

The degree of IHC staining in the tissue sections was scored independently by two pathologists who were blinded to the clinical and pathological data. Staining intensity was graded using a scale of 0–3 as follows: 0, No staining; 1, weak staining; 2, moderate staining; and 3, strong staining (11). The extent of staining was graded on a scale as follows: 0, ≤5%; 1, 6–25%; 2, 26–50%; 3, 51–75%; or 4, 76–100% according to the percentage of the section exhibiting positive staining, relative to the entire carcinoma-involved area (12,13). The intensity and extent scores were multiplied to generate the immunoreactivity score (IS; range, 0–12) for each case (12,13). β-catenin immunoreactivity was separately analyzed for the tumor center and the tumor invasive front. Specimens were rescored if there were discrepancies in the IS obtained by the two pathologists, until a consensus was reached. Membranous expression of β-catenin was classified as preserved when >80% of the cell membrane was stained; otherwise, the sample was classified as exhibiting reduced expression (14). High cytoplasmic and nuclear β-catenin expression grades were defined as >50% reactivity of the tumor cells (15).

### Statistical analysis

Statistical analysis was performed using SPSS software (version 17.0; SPSS, Inc., Chicago, IL, USA). The paired-samples t-test was used to compare the differential β-catenin expression levels between the tumor center and the tumor invasive front. The statistical significance of the associations between β-catenin expression levels and the patient clinicopathological parameters was assessed using χ^2^ tests. Kaplan-Meier survival curves were plotted to analyze the distribution of CRC-specific survival times and intergroup differences were determined using the log-rank test. A multivariate Cox regression model through a stepwise selection procedure was applied to examine the independence of the significant factors identified in the univariate analysis. Cox proportional hazards regression was used to calculate the mortality hazard ratios according to various clinicopathological features and protein markers. Two-sided P<0.05 was considered to indicate a statistically significant difference.

## Results

### Expression of β-catenin in CRC

**Figure 1:**
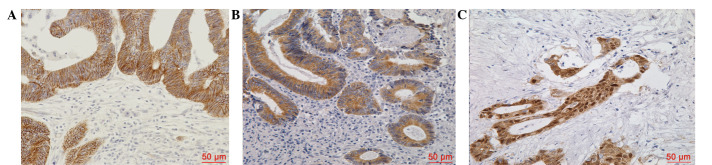
Immunohistochemical staining of β-catenin in colorectal cancer samples. (A) β-catenin membranous expression. (B) β-catenin cytoplasmic expression. (C) β-catenin nuclear expression.

**Figure 2:**
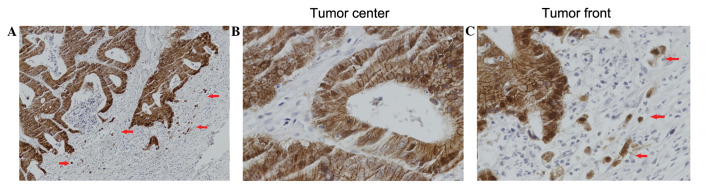
Immunohistochemical staining demonstrating differential β-catenin expression levels at the tumor center and at the tumor invasive front. (A) Overview picture demonstrating both the tumor center and the tumor invasive front. The red arrows indicate tumor budding and nuclear β-catenin expression. (magnification, ×200). (B) At the tumor center, β-catenin expression was predominant at the cell membrane. (C) At the tumor invasive front, the red arrows indicate that the isolated single tumor cells or the small clusters of tumor cells (tumor budding) had scattered from the primary mass with marked nuclear β-catenin accumulation (epithelial-mesenchymal transition phenotype; magnification, ×400).

β-catenin protein expression was evaluated in the CRC sections via IHC analysis. As shown in Fig. 1, β-catenin staining was observed predominantly at the cell membrane, and marginally in the cell cytoplasm and nucleus. Membranous β-catenin expression was identified to be significantly higher in the tumor center than at the tumor invasive front (IS: 5.36±3.812 versus 0.42±1.252, respectively; P<0.001). However, reduced membranous β-catenin expression levels in the tumor center were identified in 107 (59.1%) of the 181 patients. Nuclear β-catenin expression levels were significantly lower at the tumor center than at the tumor invasive front (IS: 0.05±0.303 versus 2.18±3.917, respectively; P<0.001) as shown in Fig. 2 and Table I. High nuclear β-catenin expression levels at the tumor invasive front were observed in 30 (16.6%) of the 181 patients.

### Correlation between β-catenin expression levels and the clinicopathological characteristics of CRC

The reduced membranous β-catenin expression levels at the tumor center were identified to be significantly associated with the occurrence of lymph node metastasis (P=0.002) and the TNM stage (P=0.002). However, no associations between reduced membranous β-catenin expression levels and age/gender at diagnosis, tumor site or size, invasion depth, presence or absence of tumor deposits, histological differentiation, or lymphatic or venous invasion were evident. In addition, no statistically significant correlations between cytoplasmic or nuclear expression levels of β-catenin and the above-mentioned clinicopathological characteristics were observed (Table II).

At the tumor invasive front, the detection of high nuclear expression levels of β-catenin was significantly correlated with lymph node metastasis (P=0.016), the TNM stage (P=0.020) and histological differentiation (P=0.006), however, not with age/gender at diagnosis, tumor site or size, invasion depth, presence or absence of tumor deposits, or lymphatic or venous invasion. In addition, high cytoplasmic expression levels of β-catenin were significantly correlated with histological differentiation (P=0.001) and tumor site (P=0.004). No statistically significant association was observed between the presence of high cytoplasmic expression of β-catenin and age/gender at diagnosis, tumor size, invasion depth, lymph node metastasis, TNM stage, presence or absence of tumor deposits, or lymphatic or venous invasion. Furthermore, no statistically significant correlation was detected between the detection of reduced membranous expression levels of β-catenin and the above-mentioned clinicopathological characteristics (Table III).

### Survival analysis

**Figure 3:**
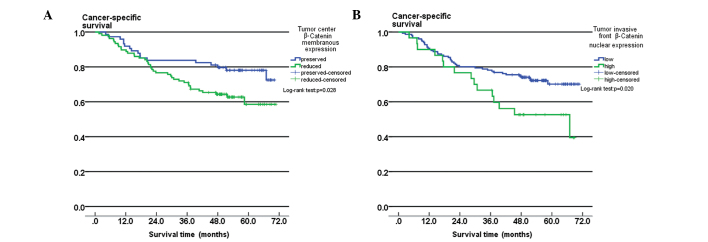
Kaplan-Meier survival analysis. (A) β-catenin membranous expression at the tumor center; preserved membranous expression vs. reduced membranous expression (P=0.028). (B) β-catenin nuclear expression at the tumor invasive front; low vs. high expression levels (P=0.020).

Patients with reduced membranous expression levels of β-catenin at the tumor center had significantly lower cancer-specific five-year survival rates (58.5%), compared with patients that exhibited preserved membranous expression of β-catenin at the tumor center (78.1%; log-rank, P=0.028; Fig. 3A). The difference in cancer-specific survival rates between patients with high-grade nuclear expression of β-catenin (five-year survival rate, 52.6%) and low-grade nuclear expression of β-catenin (five-year survival rate, 70.1%) at the tumor invasive front was also identified to be statistically significant (log-rank test, P=0.020; Fig. 3B).

Univariate analysis revealed that the T stage (P<0.001), N stage (P<0.001), TNM stage (P<0.001), the presence of lymphatic invasion (P<0.001), the presence of tumor deposits (P<0.001), reduced membranous expression levels of β-catenin at the tumor center (P=0.028) and high-grade nuclear expression of β-catenin at the tumor invasive front (P=0.020) were significant prognostic factors. However, age, gender, tumor location and size, tumor differentiation and venous invasion were not significantly associated with patient survival (Table IV).

Multivariate analysis using Cox regression analysis demonstrated that the TNM stage (P<0.001), presence of tumor deposits (P=0.001) and lymph node metastasis (P=0.026) were independent prognostic factors in CRC patients (Table IV). In addition, multivariate analysis revealed that β-catenin levels were not a significant prognostic factor.

## Discussion

Despite significant advancements in CRC diagnosis and treatment, the prognosis for patients with advanced CRC remains poor. EMT, the switch from the polarized epithelial cell phenotype to a migratory mesenchymal phenotype, is increasingly recognized as a central event during malignant tumor progression and metastasis (16,17). β-catenin maintains cell-to-cell adhesion along with E-cadherin. However, β-catenin also acts as a transcription factor in the Wnt signal transduction pathway (5). The dual role of β-catenin in cadherin-mediated cell-cell adhesion and in Wnt signaling, where it is a key effector, renders β-catenin an ideal target for analyzing the molecular basis of EMT and malignant cancer formation. The accumulation and aberrant activation of β-catenin signaling, as well as the transcription of target genes (hypothesized to contribute to various stages in tumor development) result from mutations in the adenomatous polyposis coli protein that abolish its capacity to bind β-catenin or mutations in the β-catenin phosphorylation motif at the N-terminus. The target genes include predominant regulators of EMT, for example Slug (18), which inhibits E-cadherin transcription. The release of β-catenin from cell-cell junctions following the dismantling of cell-cell adhesions, which contain E-cadherin, during EMT and the consequent activation of β-catenin-mediated transactivation are also important in EMT regulation (19).

However, the prognostic significance of β-catenin expression levels in patients with CRC remains controversial. Certain studies have shown that nuclear β-catenin expression is associated with high tumor budding and a poor prognosis (15,20,21), however, other studies did not detect this association (22–24). Additionally, a recent study revealed that β-catenin overexpression was correlated with a favorable prognosis (25).

Therefore, in the present study, the expression levels of the EMT-associated marker, β-catenin were investigated at the tumor invasive front and in the tumor center of CRC tissue specimens. Long-term clinical follow-up of the CRC patients was conducted and a large number of cases were included in the study, thus, the results are considered to be meaningful.

The levels of β-catenin protein expression in serial paraffin-embedded sections obtained from 181 human CRC samples were examined using IHC staining. The correlations between the β-catenin differential expression patterns and clinicopathological characteristics and prognosis were also assessed. The results showed that membranous β-catenin expression levels in the samples were significantly reduced at the tumor invasive front, compared with in the tumor center; however, nuclear β-catenin expression levels were significantly increased at the tumor invasive front, compared with in the tumor center. The dynamic changes in the intracellular distribution of β-catenin and the changes in tumor cell phenotype revealed a process that is reminiscent of EMT. The presence of reduced membranous expression levels of β-catenin at the tumor center was significantly associated with lymph node metastasis and the TNM stage. At the tumor invasive front, high-grade nuclear expression of β-catenin was significantly correlated with lymph node metastasis, TNM stage and histological differentiation.

Further analysis demonstrated that patients with reduced membranous expression levels of β-catenin had significantly lower cancer-specific five-year survival rates, compared with those patients exhibiting preserved membranous expression of β-catenin at the tumor center. The five-year survival rate of patients with high-grade nuclear β-catenin expression was significantly lower than that of patients with low-grade nuclear expression of β-catenin at the tumor invasive front. However, this did not persist as an independent prognostic factor following Cox multivariate analysis. The undifferentiated tumor cells at the tumor invasive front may undergo β-catenin-mediated EMT, which may result in the dissemination of tumor cells, and subsequently induce tumor invasion and metastasis.

In conclusion, the results of the present study indicate that changes in β-catenin expression levels are associated with aggressive morphological features, EMT and a poor prognosis in patients with CRC. IHC staining of β-catenin is considered to be a useful marker to predict the prognosis in CRC. However, large, well-designed prospective studies are required to further investigate the accurate prognostic significance of β-catenin.

## Figures and Tables

**Table I:** Paired sample comparison (t-test) of β-catenin expression levels between the tumor center and tumor invasive front.

	Immunoreactivity score		
	
Variable	No.	Mean ± SD	P-value
Membranous β-catenin			
Tumor center	181	5.36±3.812	<0.001
Tumor front	181	0.42±1.252	
Nuclear β-catenin			
Tumor center	181	0.05±0.303	<0.001
Tumor front	181	2.18±3.917	

P<0.05 indicates a significant difference. SD, standard deviation.

**Table II:** β-catenin expression levels at the tumor center in association with patient clinicopathological variables.

		Membranous expresssion levels			Cytoplasmic expression levels			Nuclear expression levels		
				
Variable	Total	Preserved	Reduced	P-value	Low	High	P-value	Low	High	P-value
Age (years)										
≤60	65	21	44	0.079	47	18	0.248	62	3	0.255
>60	116	53	63		92	24		114	2	
Gender										
Male	105	43	62	0.982	82	23	0.626	103	2	0.408
Female	76	31	45		57	19		73	3	
Tumor size (cm)										
≤5	94	40	54	0.635	71	23	0.675	91	3	0.714
>5	87	34	53		68	19		85	2	
Tumor site										
Colon	72	27	45	0.425	59	13	0.182	69	3	0.349
Rectum	109	47	62		80	29		107	2	
T stage										
pT1–pT2	61	30	31	0.106	52	9	0.055	60	1	0.511
pT3–pT4	120	44	76		87	33		116	4	
N stage										
pN0	108	54	54	0.002^a^	88	20	0.069	104	4	0.347
pN1–pN2	73	20	53		51	22		72	1	
TNM stage										
I–II	107	54	53	0.002^a^	87	20	0.084	103	4	0.335
III–IV	74	20	54		52	22		73	1	
Tumor deposit										
Absent	152	65	87	0.239	117	35	0.897	147	5	0.322
Present	29	9	20		22	7		29	0	
Differentiation										
Well, mod	132	57	75	0.302	98	34	0.182	127	5	0.167
Por, muc	49	17	32		41	8		49	0	
Lymph invasion										
Negative	167	70	97	0.329	128	39	0.870	162	5	0.511
Positive	14	4	10		11	3		14	0	
Venous invasion										
Negative	178	73	105	0.789	136	42	0.337	173	5	0.768
Positive	3	1	2		3	0			3	0

a Statistically significant (P<0.05).

TNM, tumor, node, metastasis; Mod, moderately differentiated; muc, mucinous adenocarcinoma; por, poorly differentiated and undifferentiated.

**Table III:** β-catenin expression levels at the tumor invasive front in association with patient clinicopathological variables.

		Membranous expression levels			Cytoplasmic expression levels			Nuclear expression levels		
				
Variable	Total	Reduced	Preserved	P-value	Low	High	P-value	Low	High	P-value
Age (years)										
≤60	65	52	13	0.151	37	28	0.080	57	8	0.248
>60	116	102	14		81	35		94	22	
Gender										
Male	105	91	14	0.482	66	39	0.438	90	15	0.330
Female	76	63	13		52	24		61	15	
Tumor size (cm)										
≤5	94	83	11	0.207	60	34	0.689	76	18	0.333
>5	87	71	16		58	29		75	12	
Tumor site										
Colon	72	58	14	0.165	56	16	0.004^a^	62	10	0.430
Rectum	109	96	13		62	47		89	20	
T stage										
pT1–pT2	61	54	7	0.354	40	21	0.939	54	7	0.188
pT3–pT4	120	100	20		78	42		97	23	
N stage										
pN0	108	91	17	0.705	73	35	0.410	96	12	0.016^a^
pN1–pN2	73	63	10		45	28		55	18	
TNM stage										
I–II	107	90	17	0.659	72	35	0.476	95	12	0.020^a^
III–IV	74	64	10		46	28		56	18	
Tumor deposit										
Absent	152	130	22	0.701	99	53	0.968	129	23	0.232
Present	29	24	5		19	10		22	7	
Differentiation										
Well, mod	132	109	23	0.120	77	55	0.001^a^	104	28	0.006^a^
Por, muc	49	45	4		41	8		47	2	
Lymph invasion										
Negative	167	143	24	0.476	110	57	0.510	139	28	0.811
Positive	14	11	3		8	6		12	2	
Venous invasion										
Negative	178	151	27	0.465	116	62	0.957	148	30	0.436
Positive	3	3	0		2	1		3	0	

a Statistically significant (P<0.05).

TNM, tumor, node, metastasis; Mod, moderately differentiated; muc, mucinous adenocarcinoma; por, poorly differentiated and undifferentiated.

**Table IV:** Univariate and multivariate analyses of survival rates in colorectal cancer patients.

		Univariate analysis		Multivariate analysis		
			
Variable	Patients (n)	Five-year survival rate (%)	P-value	HR	95% CI	P-value
Age (years)						
≤60	65	69.7	0.254			
>60	116	66.0				
Gender						
Male	105	69.9	0.151			
Female	76	64.0				
Tumor size (cm)						
≤5	94	64.2	0.814			
>5	87	70.6				
Tumor site						
Colon	72	65.2	0.266			
Rectum	109	68.3				
T stage						
pT1–pT2	61	85.7	<0.001^a^			
pT3–pT4	120	58.5				
N stage						
pN0	108	92.5	<0.001^a^	10.729	1.336–86.14	0.026^a^
pN1–pN2	73	32.3				
TNM stage						
I–II	107	93.4	<0.001^a^	0.009	0.001–0.082	<0.001^a^
III–IV	74	31.8				
Tumor deposit						
Absent	152	76.8	<0.001^a^	0.368	0.208–0.651	0.001^a^
Present	29	17.2				
Differentiation						
Well, mod	132	68.7	0.984			
Por, muc	49	62.8				
Lymph invasion						
Negative	167	70.1	<0.001^a^			
Positive	14	35.7				
Venous invasion						
Negative	178	67.9	0.086			
Positive	3	33.3				
Tumor center membranous β-catenin						
Preserved	74	78.1	0.028^a^	1.132	0.627–2.046	0.681
Reduced	107	58.5				
Tumor front nuclear β-catenin						
Low-grade	151	70.1	0.020^a^	0.708	0.384–1.705	0.268
High-grade	30	52.6				

a Statistically significant (P<0.05). The forward stepwise Cox regression approach was performed for the statistical analyses and the non-significant variables are not presented in the final table.

CI, confidence interval; HR, hazard ratio; TNM, tumor, node, metastasis; mod, moderately differentiated; muc, mucinous adenocarcinoma; por, poorly differentiated and undifferentiated.
